# The ratio model between throughput and delay based on payload transmission time in wireless blockchain network

**DOI:** 10.1038/s41598-022-19138-z

**Published:** 2022-09-12

**Authors:** Chun Shi, Chun Shan

**Affiliations:** 1 School of Electronic and Information, Guangdong Polytechnic Normal University, Guangzhou, 510633 People’s Republic of China; 2 Guangxi Key Laboratory of Multimedia Communications and Network Technology, Nanning, 530004 People’s Republic of China

**Keywords:** Electrical and electronic engineering, Computer science

## Abstract

In wireless blockchain network (WBN), it is necessary to study the impact of delay performance on blockchain technology with different data rates, because the throughput performance is not the only goal. We propose an analysis model to evaluate network performance based on both delay performance and network performance. Firstly, we separate the communication process from business process in WBN, and package the business data into the payload in a frame. Secondly, we define throughput-time $S_V$ as a ratio of throughput to data transmission rate and propose a ratio model of $S_V$ to network delay. Then, we analyze the ratio model with the parameter of payload transmission time, which is defined as a ratio of payload in a frame to data transmission rate. Using the DATA/ACK type in IEEE 802.11, we learn that the optimal payload transmission time is independent of node number and only determined by some interframe space of access mechanism and propagation time. Finally, we analyze the performance of network delay and throughput between DATA/ACK type and RTS/CTS/DATA/ACK type based on payload transmission time. The simulation data verify the validity and accuracy of the proposed model.

## Introduction

In recent years, the blockchain technology has attracted the interest from academic and industry. The wireless blockchain network (WBN) is considered to be a promising application direction, which has the natural combination of blockchain technology and Internet of Things (IoTs) and can bring about the comprehensive transmission of information for the accuracy of information^1–3^.

Since the blockchain technology mainly assumes a stable and high-throughput wired network, it’s necessary to fully study the impact of wireless network performance in WBN. The existing optimization schemes of wireless network usually aim to improve throughput performance, and cannot be directly applied to WBN. Generally, there are some performance indicators in WBN, including transaction throughput, transaction delay, communication throughput and communication delay^4,5^. In WBN, there are various performance analysis metrics and different network scenarios corresponding to different quality of service (QoS) requirements. With wide applications, there are various protocol standards and devices in IoTs. Aiming at the heterogeneous network scenarios with different data rates, we focus on two performance metrics of communication throughput and communication delay. In the following paper, throughput and delay (or network delay) mean communication throughput and communication delay, respectively.

The IEEE 802.11 protocols offer popular applications for high-speed wireless communication^6,7^. The performance analyses of WBN contain the distribution coordination function (DCF) mechanism of IEEE 802.11^8–10^. In WBN, the delay ( transaction delay + communication delay) is long due to data verification and consistency mechanism. Therefore, throughput is not the only goal. There is an upper limit on transaction throughput. The maximum transactions per second (TPS) of bitcoin and Ethereum are 7 and 20 respectively^5,8,11^. Bitcoin can only read and write fixed length data. Ethereum expands the function of blockchain and can read and write data of different lengths. Focusing on the parameter of variable data lengths, we analyze the optimization scheme of access parameters based on delay performance.

We focus on different data rates and trade-offs between throughput performance and delay performance in WBN. Normally, the throughput performance of DCF mechanism is usually the main research. Based on the throughput performance, the network delay can be calculated, which means that delay is just another expression of throughput performance. Although throughput and network delay are two main parameters of DCF mechanism, they cannot be directly used to evaluate performance of WBN. In fact, there are two problems that need to be solved. One problem is the impact of different data rates on performance evaluation in heterogeneous devices; Another problem is the independence of data transmission process and business process of blockchain technology, as well as the balanced performance of throughput and delay.

In this paper, we try to find the optimization scheme of access parameters based on delay performance. We focus on the different data rates of heterogeneous devices in WBN, and analyze the impact of variable payload length on the balance relationship between throughput and delay. From the communication perspective, we simplify the business process of WBN, and package the business data into the payload in the medium access control (MAC) frame during performance analysis. Also we introduce the parameter of payload transmission time, which can cover the difference of multiple data transmission rates. Then, we analyze the different increase rates of throughput and network delay based on the payload transmission time. We want to find a balance that delay performance can determine access parameters and obtain a higher increase rate of throughput than that of network delay.

The rest of the paper is organized as follows. We first introduce data process and business process of blockchain technology in “Preliminaries” section. We then briefly describe the state of the art of in “State of the art” section. In “Ratio model based on payload transmission time” section, we propose a ratio model of throughput to network delay, followed by the analysis of the optimal payload transmission time and the limitation of request-to-send/clear-to-send (RTS/CTS) type based on the payload transmission time. Simulation, numerical results and analysis are shown in “Simulation and analysis” section. Finally, “Conclusion” section concludes the paper.

## Preliminaries

In this section, we first introduce the data exchange process in DCF mechanism. Then, we give the business process of blockchain technology and some simplifications.

### Data exchange process in DCF

**Figure 1:**
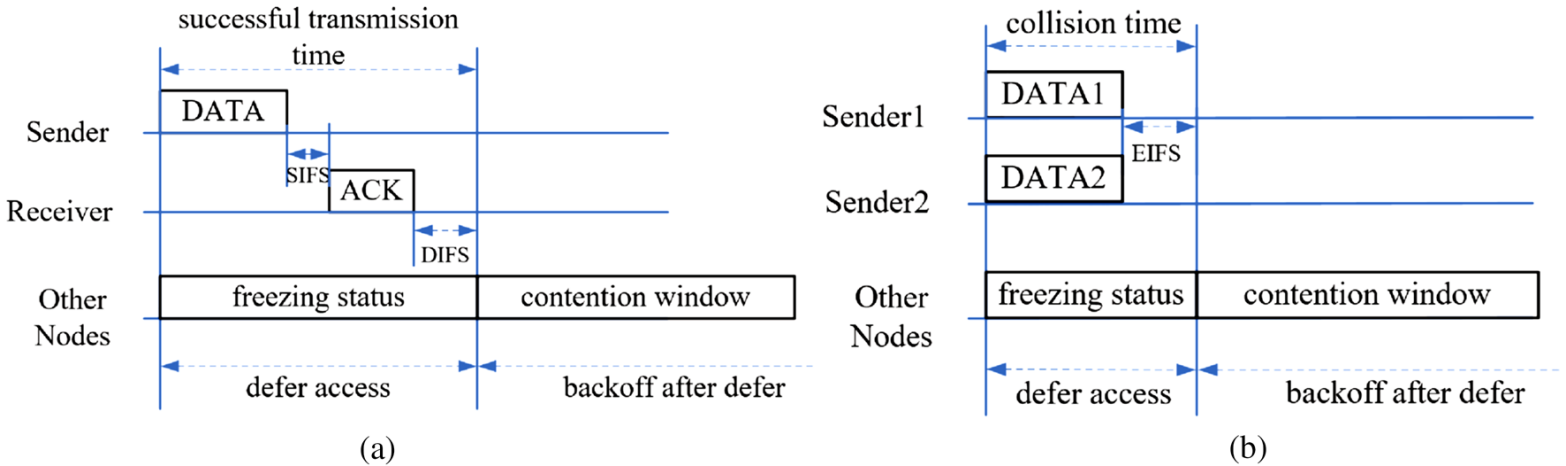
Successful transmission process and collision process based on the DATA/ACK type. The successful transmission time is shown in (**a**) and the collision time is shown in (**b**).

The main mechanism is the DCF mechanism in IEEE 802.11. Before sending a data frame in DCF mechanism, the source node can use a handshaking technique, known as RTS/CTS, to reserve the channel. The usage of RTS/CTS frames is intended to reduce the collision time before sending long data frame. The DCF mechanism provides two types of data exchange: RTS/CTS/DATA/ACK type and DATA/ACK type. We start our analysis with DATA/ACK type as shown in Fig. 1, in which the data frame length directly affects network performance of throughput and network delay.

During the DATA/ACK type, nodes sense the states of channel before sending data frames. If the channel is idle, nodes with a frame will decrease one value of the backoff window. When the value of backoff window is zero, the tagged node begins to send a frame. If the tagged node gets the ACK frame, it will initiate a new frame transmission as shown in Fig. 1a. If the tagged node doesn’t receive an ACK frame, there may be a collision in the channel in Fig. 1b. If other nodes detect a busy channel state, they freeze all activity and wait for the duration of a successful data transmission time or collision time.

In the decrease process of backoff window, there are some activities of other nodes in the channel, like idle event, collision event and successful transmission event. If there is a successful transmission, the tagged node needs to stop the decrease process of backoff window for an additional period of time named distributed interFrame space (DIFS) ($T_{DIFS}$). For collisions in the channel, the tagged node will wait for an additional period of time named extended interFrame space (EIFS) ($T_{EIFS}$) beyond the duration of collision events. The additional time of $T_{EIFS}$ and $T_{DIFS}$ will increase the network delay. The more activities other nodes have, the longer the network delay will be.

To decrease collision probability, nodes should have large and different backoff windows. Because the backoff window is randomly selected from the contention window (CW), the range of CW is an important parameter. $W_{max}$ denotes the maximum value of CW and $W_{min}$ is the minimum value of CW. It is a key step to adjust sizes of CW from $W_{min}$ to $W_{max}$. The CW size ($W_i$) of the *i*th data transmission increases in an exponential mode until reaching the maximum value $W_{max}$.

1 $$\begin{aligned} W_i = {\left\{ \begin{array}{ll} W_m=W_{max}-1 &{} {\text {i}}={\text {m}},\ldots ,{\text {K}}-1.\\ W_{min}-1 &{} {\text {i}}=0 \\ 2*(W_{i-1}+1)-1 &{} {\text {i}}=1,\ldots ,{\text {m}}-1 \end{array}\right. } \end{aligned}$$

where *m* is the maximum backoff stage that can be used to determine the sizes of CW. *K* is the maximum number of transmission attempts ($K{\ge }m$).

The data exchange of DCF mechanism is a basic model of reliable data transmission type, which has no other additional control frames. It’s difficult to assess the performance of throughput and network delay with different data transmission rates and variable payload lengths in networks with DATA/ACK type, due to lack of appropriate parameters. Based on a parameter of payload transmission time, we propose a ratio model to analyze the network performance, and further compare the performance of throughput and network delay between DATA/ACK type and RTS/CTS/DATA/ACK type.

### Business process of blockchain technology

We introduce lightweight nodes (LNs), full nodes (FNs) and access Point (AP)^3,9^. LNs are storage and power-constrained nodes that can issue transactions. FNs are nodes with enough computing power and storage space that generate new blocks for recording transactions.

The main steps of the consensus process in WBN can be summarized as follows:

1. The LNs broadcast transactions to FNs.
2. After receiving the new transactions, FNs generate a new block and send it to AP.
3. After getting the new block, the AP broadcasts it to all FNs.
4. The other FNs insert the new block into their local ledgers.
5. If AP and most of the FNs have the correct new block, the new block and its transactions consensus are completed.

We can see that LNs, FNs and AP have some unique data to process before transmitting different messages, which is the business feature of blockchain technology. We separate the data transmission process from the business process of blockchain technology. This means that the data transmission process only includes sending and receiving data. Transaction generation, storage and validation are not in the process of data transmission. These messages transmitted by LNs, FNS and AP are payloads in MAC frames. These payloads are transparent to the data transmission processes. We mainly analyze the performance of data transmission process and propose a new performance evaluation method that compromises throughput and delay in WBN.

## State of the art

We define the network delay (or delay) as the time interval between the time when the packet reaches the head of the transmission queue and contends for the channel, and the time when the node receives the corresponding ACK frame. The throughput is defined as the payload transmitted during the network delay. Generally, the throughput performance of access mechanism is the only goal and there are two ways to improve throughput performance: one is to optimize the access mechanism for the lower collision probability of data transmission; The other is to increase the duration of data transmission.

An analytical model^12^ based on Markov chain model is proposed to analyze the saturation performance with access parameters like CW, which has been verified with a fixed payload (8184 bits) in a frame. Two steady-state operating points for the unsaturated networks and the saturated networks are proposed to analyze the performance with some access parameters, such as CW and cutoff (backoff) phase^13^. An adaptive access mechanism is analyzed^14^, which presents an optimal size of CW about the node number. These works mainly focus on analyzing and optimizing access parameters to reduce collision probability of data transmission.

Some technologies, like methods of frame aggregation and transmission opportunity (TXOP), are used to obtain the maximum throughput. These methods of frame aggregation and TXOP can minimize the communication overhead of control frames and increase the duration of data transmission in the channel. These frame aggregation methods allow aggregating MAC frames into larger ones to be sent within a single TXOP. The TXOP methods allow node to sequentially transmit multiple frames within a parameter $TXOP_{limit}$. The frame aggregation and TXOP can interact with each other and increase the throughput performance^7,15^. The two-level frame aggregation, the optimal length of frame aggregation and aggregation efficiency are analyzed under some conditions, such as, different data rates or BERs^16–18^. However, the network delay is not an independent metric to evaluate network performance, but just another expression of throughput performance, which can be calculated based on the throughput performance. The parameter $TXOP_{limit}$ settings and TXOP sharing mechanism are analyzed to meet the needs of different services and maximize the throughput of differentiated traffic^19^. The method of Block ACK is analyzed to reduce the exchange of control frames^20–22^.

In order to improve throughput, access parameters are usually optimized, such as reducing collision probability or prolonging payload transmission time. In the simulation of DATA/ACK type, 33bytes, 40bytes, 1000bytes, 1023bytes, 1500bytes and other regular effective data lengths are usually adopted^12,14,21,23^. Although these chosen data are used to verify the correctness of the optimization schemes in throughput performance optimization, their lengths have no clear meaning.

In WBN, the delay is more important and needs deeper study. However, there is no effective optimization scheme of access parameters based on delay performance. Queuing delay and consensus delay are analyzed^10^, and there is no distinguish between business content and data transmission process. Due to the mixing of business process of blockchain technology and data transmission process, the complexity of analysis is increased. The communication delay between lightweight IOT devices and blockchain nodes is analyzed^24^. However, there are few methods for wireless delay optimization. Delay is usually another calculation of throughput performance. That is, after obtaining the optimal throughput, relevant access parameters are used to calculate delay. In WBN, the main method also focuses on throughput performance optimization. Based on the throughput performance and delay performance, the scalability limitation of blockchain is studied on block size and block interval^4,25,26^. The deferred execution mechanism is analyzed to get stable sequential blocks for better throughput performance^25^. Four block access control methods are studied, and a discard strategy is proposed to remove forked blocks^3^. The consensus mechanisms of PoW and PoS are studied^27–29^.

If the transmitted data is large, RTS/CTS frames can be used for channel reservation. The use of RTS/CTS frames can decrease the probability of data retransmission for higher throughput^5,10^, and the normal reason is the large collision duration of data frame, which means the used RTS/CTS frames have short duration of transmission if collisions occur. With the increased data transmission rates, the collision durations caused by data frame decrease and the overhead of RTS/CTS frames will maintain a high percentage in the data transmission cycle. Based on the simulation experiment, the relationship between throughput performance and RTS threshold is analyzed^30^; The dynamic adjustment of RTS threshold is analyzed^31^ by conducting an experimental characterization of RTS/CTS as a function of packet size, transmission rate, and network contention. The calculations of RTS threshold in bits are proposed^32,33^, and the effects of successful transmission probability, control frame rate and data transmission rate on RTS threshold are analyzed. The function of RTS/CTS frames can also be applied together with other technologies, such as compressed sensing technology^34^. Relevant application scenarios include frequency domain^35^, linear ad hoc network^36^, high-speed train^37^, data privacy and security^38–41^. For different transmission rates, the RTS threshold defined in time will be more fit. Once the protocol is chosen, the control frame rate and collision probability can also be considered as constants. Therefore, the analysis based on payload transmission time will be more valuable.

Once the protocol or optimization scheme is determined, the access parameters will also be fixed. With the increase of payload length and different data transmission rates, it is complex to analyze and evaluate the network performance. How does the payload length in a frame affect the network performance? Is the throughput increase rate equal to the network delay increase rate? A single parameter of throughput or delay cannot be used to evaluate the optimization scheme. Based on the payload transmission time, we propose an evaluation method combining throughput and delay, then the effectiveness and correctness of the evaluation are analyzed and verified.

## Ratio model based on payload transmission time

We assume that every node is saturated, which means each node always has data to send. We also assume nodes transmit with the DATA/ACK type. Firstly, we propose the ratio model of throughput-time to network delay. Secondly, we analyze the optimal payload transmission time based on the ratio model. Lastly, we compare the network performance between RTS/CTS/DATA/ACK type and DATA/ACK type.

### Ratio model of throughput to network delay

#### Analysis of network delay

According to the data exchange of DATA/ACK, we have the successful transmission time $T_S$ and collision time $T_C$ as

2 $$\begin{aligned} \left\{ \begin{array}{l} {T_S=T_{HEAD}+t_{DATA}+2{\sigma }+T_{SIFS}+T_{ACK}} \\ {T_C=T_{HEAD}+t_{DATA}+{\sigma }+T_{SIFS}} \end{array} \right. \end{aligned}$$

where ${\sigma }$ is the propagation time. $T_{SIFS}$ denotes the duration of the Short InterFrame Space (SIFS). $t_{DATA}$ is the payload transmission time in a frame, which is the main interest in this work. $T_{HEAD}$ and $T_{ACK}$ denote transmission time of frame headers and transmission time of ACK frame respectively. There are two parts in the frame header length: One part is the PHYsical header length $T_{HEAD}^{PHY}$, and the transmission rate is the same as the control frame rate. Another is the Medium Access Control header length $T_{HEAD}^{MAC}$, using the same rate as data transmission rate. The time of frame header is

3 $$\begin{aligned} T_{HEAD}=T_{HEAD}^{PHY}+T_{HEAD}^{MAC} \end{aligned}$$

where $T_{HEAD}^{PHY}$ is determined by the control frame transmission rate. Since the control frame transmission rate is basically fixed, $T_{HEAD}^{PHY}$ is usually constant. The data transmission rate will take different values according to the network conditions. Therefore, the value of $T_{HEAD}^{MAC}$ is dynamic. For given the data transmission rate, frame header length $T_{HEAD}$ is fixed, and then the length of the transmitted frame can easily be calculated with values of the payload length.

We analyze states of busy channel and have

4 $$\begin{aligned} p= & {} 1-(1-\tau )^{N-1} \nonumber \\= & {} [(N-1)\tau (1-\tau )^{N-2}] \nonumber \\&+[1-(1-\tau )^{N-1}-(N-1)\tau (1-\tau )^{N-2}]\nonumber \\= & {} p_S+p_C \end{aligned}$$

where $\tau$ is the attempt probability that a node transmits in randomly chosen slot time. *p* is the conditional collision probability that means probability of a busy channel. $p_C=[1-(1-\tau )^{N-1}-(N-1)\tau (1-\tau )^{N-2}]$ and $p_S=[(N-1)\tau (1-\tau )^{N-2}]$ are probabilities of collision and successful data transmission respectively. With the conditional collision probability *p*, the CW minimum value $W_0$, the maximum transmission number *K*, the backoff stage *m* and the backoff multiplier factor $\lambda$, we have

5 $$\begin{aligned} \tau ^{-1}=\frac{(1-p)W_{0}(1-({\lambda }p)^m)}{2(1-p^K)(1-{\lambda }p)}+\frac{{\lambda }^mW_{0}(p^m-p^K)}{2(1-p^K)}-\frac{1}{2} \end{aligned}$$

  The calculations of *p* and $\tau$ can establish a fixed point formulation by using numerical techniques. Then, the values of $p_C$ and $p_S$ can also be calculated.

Since nodes randomly choose numbers of backoff window in the range of CW, the mean sizes of backoff window are important for the decrease process of backoff window. Let $E[U^{(j)}]$ denote the mean sizes of backoff window and it means the average waiting slot numbers before the *j*th transmission attempt. We have

6 $$\begin{aligned} E[U^{(j)}] = {\left\{ \begin{array}{ll} \frac{{\lambda }^j \cdot W_0-1}{2} &{} {\text {for}}\,{\text {j}}=0,\ldots ,{\text {m}}-1, \\ \frac{{\lambda }^m \cdot W_0-1}{2} &{} {\text {for}}\,{\text {j}}={\text {m}},\ldots ,{\text {K}}-1. \end{array}\right. } \end{aligned}$$

where $W_0$ is the initial CW size.

We start from the performance of network delay, and get the calculation of network delay *E*[*D*] as

7 $$\begin{aligned} E[D]\;=\;& {} (p_S \cdot T_{DIFS}+p_C \cdot T_{EIFS} + T_{SLOT}){\sum ^{K-1}_{i=0}\sum ^{i}_{j=0}\eta p^i E[U^{(j)}]} \nonumber \\&+\,T_S\cdot {(p_S{\sum ^{K-1}_{i=0}\sum ^{i}_{j=0} \eta p^iE[U^{(j)}]}+1)} \nonumber \\&+\, T_C\cdot {(p_C{\sum ^{K-1}_{i=0}\sum ^{i}_{j=0}\eta p^i E[U^{(j)}]}+\sum ^{K-1}_{i=0}i \cdot \eta p^i)} \end{aligned}$$

where $\eta =(1-p)(1-p^K)^{-1}$. $T_{SLOT}$ is the slot time. We define ${(\sum ^{K-1}_{i=0}{\sum ^i_{j=0}}{\eta p^i}{E[U^{(j)}]}})$, $({\sum ^{K-1}_{i=0}i \cdot \eta p^i})$ and $(p_S \cdot T_{DIFS} + p_C \cdot T_{EIFS} + T_{SLOT})$ as $\pi _{1}$, $\pi _{2}$ and $\beta _{1}$ respectively. $\pi _{1}$ is the mean value of slot that the tagged node needs to wait for to complete a data transmission with the parameter of conditional collision probability *p* and transmission attempts *K*. $\beta _{1}\cdot \pi _{1}$ represents the idle channel duration due to no data transmission. $T_S\cdot {(p_S\cdot \pi _{1}+1)}$ means the duration of the successful data transmissions. $T_C \cdot {(p_C\cdot \pi _{1}+\pi _{2})}$ means the duration of the collisions in the channel.

We have network delay as

8 $$\begin{aligned} E[D]\;=\;{T_S(p_S \cdot \pi _1+1)+T_C(p_C \cdot \pi _1+\pi _2)}+\beta _1 \cdot \pi _1 \end{aligned}$$

  According to a steady network with known node numbers, these values of parameters $\pi _1,\ \pi _2$ and $\beta _1$ are normally fixed, and these values of parameters can be assumed to be constant for normal analysis. Other parameters, like $T_{HEAD}$, $T_{SIFS}$, $T_{DIFS}$ and $T_{ACK}$, are predefined and cannot be optimized. $t_{DATA}$ is the payload transmission time and its value is equal to the ratio of payload to data transmission rate.

To simplify the analysis, we assume that $t_{DATA}$ is a continuous variation. Based on the variable parameter of $t_{DATA}$, we rewrite network delay as

9 $$\begin{aligned} E[D\;]= \;& {} {t_{DATA}}\cdot {(1+(p_S+p_C)\cdot \pi _1+\pi _2)}+\beta _2 \nonumber \\= & {} {t_{DATA}}\cdot {(1+p\pi _1+\pi _2)}+\beta _2 \end{aligned}$$

where $$\beta _2=(T_{HEAD}+T_{SIFS}+\sigma )\cdot (1+p\pi _1+\pi _2)+(\sigma +T_{ACK})(p_S\pi _1+1)+T_{DIFS}(p_C\pi _1+\pi _2)+\beta _1\cdot \pi _1$$.

In (9), we find that there is a linear relationship between network delay *E*[*D*] and the payload transmission time $t_{DATA}$. If $t_{DATA}=0$, no packet is sent successfully, and the value of network delay is not equal to ’0’ for some control frames exchange. The value of network delay increases with the increase of $t_{DATA}$. The short time of $t_{DATA}$ means the short transmitted payload length. The long time of $t_{DATA}$ means the long payload length. Only based on the performance of network delay, we cannot conclude the optimal payload length and the payload transmission time $t_{DATA}$. Then, we further analyze throughput performance.

#### Ratio of throughput to data rate

According to^14^, we have a calculation of throughput as

10 $$\begin{aligned} S= \frac{E[P](1+{p_S}\cdot {\pi _1})}{E[D]} \end{aligned}$$

where *E*[*P*] is the transmitted payload in a frame. The $E[P](1+{p_S}\cdot {\pi _1})$ represents all successfully transmitted data. The *E*[*D*] means the network delay. The calculation of throughput in (10) is closely connected with network delay. However, $E[P](1+{p_S}\cdot {\pi _1})$ only represents the received payload and has nothing to do with data transmission rate, which means that it does not reflect any information related to data transmission rate. The difference of throughput in (10) caused by multiple data transmission rates cannot be analyzed carefully.

To analyze the throughput performance with different data transmission rates, we define a parameter of throughput-time $S_V$ that means a ratio of throughput to data transmission rate. Let *V*(*t*) denote the data transmission rate. We have

11 $$\begin{aligned} S_V\;=\;\frac{S}{V(t)} \end{aligned}$$

Based on (11), we further have

12 $$\begin{aligned} S_V\;=\;& {} \frac{t_{DATA}{(1+{p_S}\cdot {\pi _1})}}{E[D]} \nonumber \\= \,& {} \frac{t_{DATA}\cdot {(1+{p_S}\cdot {\pi _1})}}{{t_{DATA}}\cdot {(1+p \cdot \pi _1+\pi _2)}+\beta _2} \end{aligned}$$

  For given access mechanism, $S_V$ is a function of payload transmission time $t_{DATA}$ in (12), which means the sum of the total time used to transmit the payload during the mean value of network delay. The throughput-time performance increases as the $t_{DATA}$ increases in a reasonable values range. The maximum value of $S_V$ is approximately equal to $\frac{1+p_S\cdot \pi _1}{1+p\cdot \pi _1+\pi _2}$ with large $t_{DATA}\ (t_{DATA} {\gg } 0)$. The larger the $t_{DATA}$ value is, the longer the throughput-time performance has. The large $t_{DATA}$ value means long payload length, which is also limited by memory space and is not suitable for practical application. Because of the linear relationship between *S* and $S_V$ ($S=S_V*{V(t)}$), we do not strictly distinguish the performance between the throughput and the throughput-time in this paper.

#### Ratio model of throughput-time to network delay

Combined with analyses of network delay in (9) and throughput-time $S_V$ in (12), there is only one variable parameter $t_{DATA}$ in throughput-time and network delay. Then, we further analyze the relationship between the increase rate of $S_V$ and the increase rate of network delay with the increase of payload transmission time.

We define a ratio model of throughput-time $S_V$ to network delay *E*[*D*] as

13 $$\begin{aligned} F\;=\;\frac{S_V}{E[D]} \end{aligned}$$

  In (13), we propose an analysis model considering throughput-time and network delay, which is a new evaluation metric. In this evaluation metric, network delay and throughput-time (throughput) play the same important function, and there is only one variable parameter $t_{DATA}$. By establishing the analysis model in (13), we further analyze the throughput-time $S_V$ and network delay with parameter of payload transmission time. Combined with (9) and (12), we can have

14 $$\begin{aligned} F(t_{DATA})\;=\;\frac{t_{DATA}(1+p_s\cdot \pi _1)}{(t_{DATA}(1+p\cdot \pi _1+\pi _2)+\beta _2)^2} \end{aligned}$$

  According to the analysis model of (14), there is only one variable parameter $t_{DATA}$ in a steady-state network. If the data exchange protocol is determined, all access parameters are known, and the payload transmission time will be the main parameter to determine the throughput-time and network delay performance. We will further analyze the different increase rates between throughput-time and network delay with the variable parameter $t_{DATA}$.

### Optimal payload transmission time

The analysis model in (14) has two main questions to be answered:

- Question One: When the payload transmission time $t_{DATA}$ changes, is there a balance between the high increase rate of throughput-time $S_V$ and the low increase rate of network delay? That is, whether there is a turning point between the increase rate of throughput-time and that of network delay. We try to find the range of parameter $t_{DATA}$ that has higher increase rate of throughput than that of network delay, and then we can evaluate the performance with low network delay and high throughput.
- Question Two: Does the balance point have practical values? If the payload transmission time in a frame is too long or too short, it cannot be used in practical applications. We further analyze the effect of payload transmission time on throughput and network delay performance.

#### Trend analysis of ratio function

With an assumption of continuous values of $t_{DATA}$, we can get a first-order derivative of $F(t_{DATA})$ as

15 $$\begin{aligned} F'(t_{DATA})=-(t_{DATA}-g)\cdot {G(t_{DATA})} \end{aligned}$$

where $$G(t_{DATA})=\frac{(1+p_s\cdot \pi _1)(1+p\cdot \pi _1+\pi _2)}{(t_{DATA}(1+p\cdot \pi _1+\pi _2)+\beta _2)^3}>0$$ and $$g=\frac{\beta _2}{(1+p\cdot \pi _1+\pi _2)}>0$$.

Once the data exchange protocol is determined, the parameters of $G(t_{DATA})$ and *g* can also be considered as constants, and there is only one variable parameter $t_{DATA}$ in (15). Obviously, $F'(t_{DATA})$ is continuous with the assumption of continuous $t_{DATA}>0$. We have

16 $$\begin{aligned} \left\{ \begin{array}{ll} {F'(t_{DATA})>0 \ \ \ \ \ \ \ (0<t_{DATA}< g)} \\ {F'(t_{DATA})<0 \ \ \ \ \ \ \ (t_{DATA}>g)} \end{array} \right. \end{aligned}$$

  According to (16), the values of $F'(t_{DATA})$ are larger than ’0’ if $t_{DATA}<g$ and the values of $F'(t_{DATA})$ are smaller than ’0’ if $t_{DATA}>g$. We have a conclusion that $F(t_{DATA})$ can reach the maximum value when $t_{DATA}=g$. There is a balance between the increase rate of $S_V$ and the increase rate of network delay with increase of $t_{DATA}$. When values of $t_{DATA}$ are smaller than *g*, the increase rates of $S_V$ will be higher than that of network delay and it’s better to increase the payload transmission time $t_{DATA}$ to get better throughput performance. When values of $t_{DATA}$ are larger than *g*, the increase rates of $S_V$ will be lower than that of network delay, which means that the increase rate of network delay will be higher than that of the $S_V$ with increased $t_{DATA}$ although the throughput may have larger values.

In (13), we present a performance analysis model considering both throughput and network delay. In this model, we obtain a balance point that the increase rate of throughput is higher than that of delay. By selecting values of payload transmission time, we can obtain the network delay and the throughput for given an access mechanism. The network delay is an important parameter that can be used to evaluate the network performance. For ’Question One’ as described above, we can answer: When the payload transmission time $t_{DATA}$ changes, there is a balance between the large increase rate of throughput-time $S_V$ and the low increase rate of network delay.

#### Payload transmission time of DATA/ACK type

Based on parameters of DATA/ACK type in IEEE 802.11, we can get an approximate calculation of *g* as

17 $$\begin{aligned} g\;=\;& {} \frac{\beta _2}{(1+p\cdot \pi _1+\pi _2)} \nonumber \\\approx & {} (T_{HEAD}+\sigma +T_{SIFS}+T_{DIFS})+\frac{p \cdot T_{EIFS}+T_{SLOT}}{p+\frac{1+\pi _2}{\pi _1}}\nonumber \\\approx & {} T_{HEAD} + T_{SIFS} + T_{DIFS} + T_{EIFS} + \sigma \end{aligned}$$

  According to (17), the optimal payload transmission time is independent of the number of nodes, and its value is determined by the access mechanism parameters and propagation time. The SIFS, DIFS and EIFS have an effect on the calculation of payload transmission time *g*. With multiple data rates, the optimal payload transmission time keeps the same, although the optimal values of frame length are different. For given data transmission rate *V*(*t*), we further calculate the optimal payload length $L_{payload}^{optimal}$ as

18 $$\begin{aligned} L_{payload}^{optimal}=g\cdot {V(t)} \end{aligned}$$

  In the calculation of payload length (18), the data transmission rate *V*(*t*) is high and will be higher, although the payload transmission time *g* is constant and short. The higher the data rate is, the longer the payload length will be. We do not discuss the longer payload length and long propagation time in this work. Obviously, the optimal value of *g* is not too large based on the parameters of IEEE 802.11, and the frame length is not too long for some data transmission rates. For ’Question Two’ as described above, we can answer: For some data transmission rates that are not very high, the optimal payload transmission time can be used in practical applications.

### Analysis between RTS/CTS/DATA/ACK and DATA/ACK

#### Analysis based on payload transmission time

Based on the payload transmission time, we further analyze the performance of RTS/CTS/DATA/ACK exchange type. We firstly rewrite the successful transmission time $T_S^{DATA}$ and collision time $T_C^{DATA}$ of DATA/ACK exchange type

19 $$\begin{aligned} \left\{ \begin{array}{l} {T_S^{DATA}=T_{HEAD}+t_{DATA}+2{\sigma }+T_{SIFS}+T_{ACK}} \\ {T_C^{DATA}=T_{HEAD}+t_{DATA}+{\sigma }+T_{SIFS}} \end{array} \right. \end{aligned}$$

We conclude the successful transmission time $T_S^{RTS}$ and collision time $T_C^{RTS}$ of RTS/CTS/DATA/ACK exchange type as

20 $$\begin{aligned} \left\{ \begin{array}{l} {T_S^{RTS}=T_{RTS}+3T_{SIFS}+T_{CTS}+T_{HEAD}+t_{DATA}}\\ {\ \ \ \ \ \ \ \ \ \ +4{\sigma }+T_{ACK}} \\ {T_C^{RTS}=T_{RTS}+{\sigma }+T_{SIFS}} \end{array} \right. \end{aligned}$$

  $E[D]^{DATA}$ and $E[D]^{RTS}$ denote the network delays of DATA/ACK type and RTS/CTS/DATA/ACK type respectively. According to network delay calculation in (8) and definitions of successful transmission time and collision time in (19) and (20), we can learn that there are some differences between $T_S^{RTS}$ ($T_C^{RTS}$) and $T_S^{DATA}$ ($T_S^{RTS}$), and they are the main parameters in the network delay calculation. Given the same payload length in a frame, we can further calculate the value between $E[D]^{DATA}$ and $E[D]^{RTS}$. We have

21 $$\begin{aligned} H(D)=E[D]^{RTS}-E[D]^{DATA} \end{aligned}$$

where *H*(*D*) means the errors between network delay $E[D]^{RTS}$ of RTS/CTS/DATA/ACK type and network delay $E[D]^{DATA}$ of DATA/ACK type. $L_{payload}^{RTS}$ and $L_{payload}^{DATA}$ represent the payload length of RTS/CTS/DATA/ACK type and the payload length of DATA/ACK type.

Let $L_{payload}^{RTS}=L_{payload}^{DATA}$, and the error of *H*(*D*) comes from the difference of data exchange type between the DATA/ACK type and RTS/CTS/DATA/ACK type. With above analyses in Eqs. (8), (19) and (20), we further have

22 $$\begin{aligned} H(D)\;=\;& {} {(T_S^{RTS}-T_S^{DATA})(p_S\cdot \pi _1+1)} \nonumber \\&{+(T_C^{RTS}-T_C^{DATA})(p_C \cdot \pi _1+\pi _2)}+\beta _1 \cdot \pi _1 \end{aligned}$$

where $${(T_S^{RTS}-T_S^{DATA})=T_{RTS}+2T_{SIFS}+2{\sigma }+T_{CTS}}$$ and $${(T_C^{RTS}-T_C^{DATA})=T_{RTS}-T_{HEAD}-t_{DATA}}$$.

With analysis in (22), we find that ${(T_S^{RTS}-T_S^{DATA})}$ only contains some values of control frames and has nothing to do with the variable parameter $t_{DATA}$. ${(T_C^{RTS}-T_C^{DATA})}$ is a negative linear function of variable parameter $t_{DATA}$ with a fixed control frame transmission time $T_{RTS}$ and header domain of frame. Let $H(D)=0$, and we can have a value of payload transmission time $h_t$ as

23 $$\begin{aligned} h_t\;=\;& {} (T_{RTS}-T_{HEAD})\nonumber \\+ & {} \frac{(T_{RTS}+2T_{SIFS}+2{\sigma }+T_{CTS})(p_S{\pi _1}+1)+{\beta _1}{\pi _1}}{p_C{\pi _1}+\pi _2} \end{aligned}$$

We further rewrite (23) as

24 $$\begin{aligned} h_t&{\approx } (T_{RTS}+T_{DIFS}-T_{HEAD})+\frac{T_{SLOT}}{p_C}\nonumber \\&\quad +\,(T_{RTS}+2T_{SIFS}+2{\sigma }+T_{CTS}+T_{DIFS})\frac{p_S}{p_C} \end{aligned}$$

  With equations of (22) and (24), if $t_{DATA}>h_t$, we can infer $H(D)<0$, which means $E[D]^{RTS}<E[D]^{DATA}$. For large payload transmitted in a frame, it’s helpful to reduce the network delay by using RTS/CTS frames before data frame transmission. If $t_{DATA}<h_t$, we can have $H(D)>0$, which means $E[D]^{RTS}>E[D]^{DATA}$. If the payload transmission time is shorter than $h_t$, the DATA/ACK exchange type is more effective than the RTS/CTS/DATA/ACK exchange type due to the shorter network delay. In this case, the exchange time of control frames (RTS/CTS frames) occupies large percentage in a data transmission cycle, and the payload transmission time is too short. We conclude as

25 $$\begin{aligned} \left\{ \begin{array}{ll} {t_{DATA}>h_t \ \ \ {\Rightarrow } \ \ E[D]^{RTS}<E[D]^{DATA}} \\ {t_{DATA}\ {\le }\ h_t \ \ {\Rightarrow } \ \ E[D]^{RTS}\ {\ge }\ E[D]^{DATA}} \end{array} \right. \end{aligned}$$

  In (25), we analyze ranges of payload transmission time based on delay performance between RTS/CTS/DATA/ACK type and DATA/ACK type. As above analyses, the value of $h_t$ is an important threshold for determining the usage of RTS/CTS frames. According to (24), we can learn that $h_t$ contains some control frames transmission time and ratio of successful transmission probability to collision probability. Normally, the transmission time of these control frames and interframe spaces are fixed for any network sizes. The successful transmission probability and collision probability, however, vary with different network sizes. The analysis in (24) shows a more accurate RTS threshold than that in^32,33^.

In this section, we analyze the ratio model of throughput-time to network delay based on the payload transmission time, which eliminates the difference of data transmission rate and can effectively evaluate the performance of throughput and network delay. The ratio model is a convex function, and its maximum value means that the increase rate of throughput is higher than that of the network delay. The optimal payload transmission time is short and the corresponding payload length can be used in practical application. We further analyze the network performance between RTS/CTS/DATA/ACK type and DATA/ACK type based on the payload transmission time. Using the ratio model, we can optimize the access scheme in WBN and adjust the access parameters according to the delay performance.

### Procedures of payload transmission time in WBN


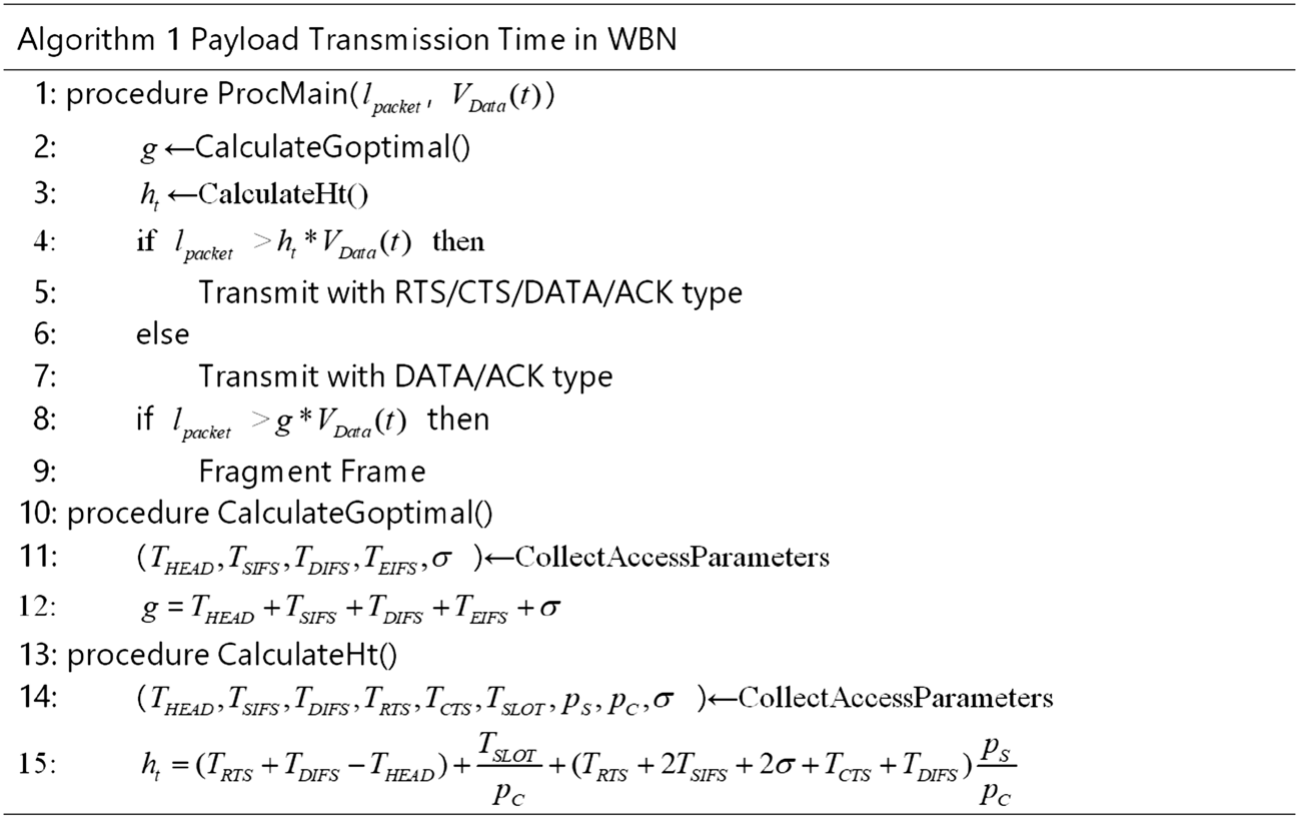


## Simulation and analysis

### Results of payload transmission time

We verify the ratio model of $S_V$ to network delay in the OPNET modeler. Each node always has data to send and exchanges the data exchange with the DATA/ACK types. The characteristic of physical layer is the direct sequence spread spectrum. In this section, we set the transmission rate of control frames as 1 Mbps. The transmission rates of data frames are 5.5 Mbps and 11 Mbps. We give the analysis results at 11 Mbps. Based on (17), we can obtain $g \approx 637\,{\upmu }\mathrm{s}$, and the payload length is equal to 876 Bytes at 11 Mbps data rate.

**Figure 2:**
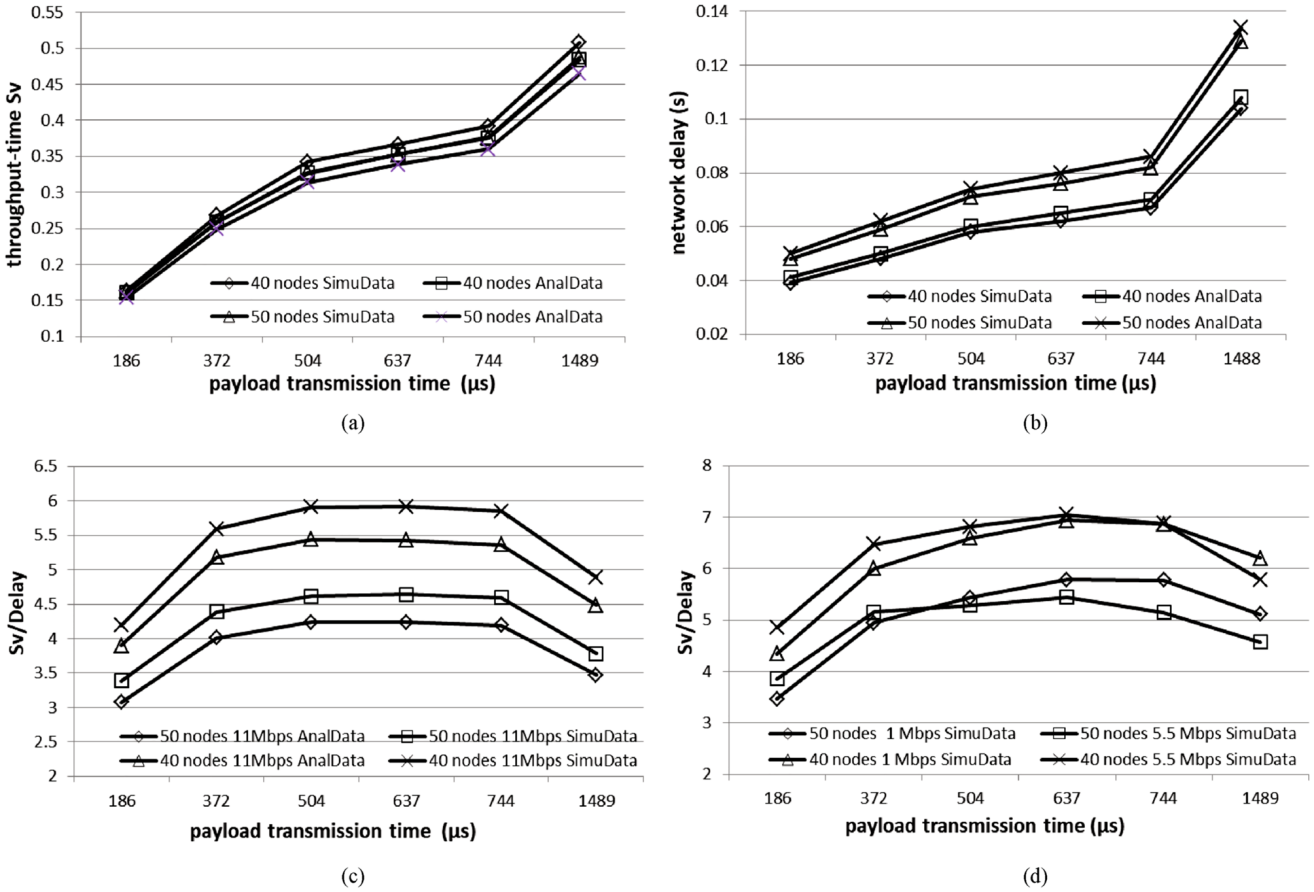
Network performance with different payload transmission time at data rate 11 Mbps. ’SimuData’ means simulation results; ’AnalData’ means analysis results. The network is composed of 40 nodes and 50 nodes. The simulation data of throughput-time performance and network delay performance are close to the analysis data in (**a**,**b**). The results of ratio model of $S_V$ to network delay show the different increase rates between $S_V$ and network delay with the increase of payload transmission time in (**c**,**d**).

We select 1023 Bytes as a referential payload length in a frame^12^, which is 744 ${\upmu }\mathrm{s}$ for 11 Mbps data transmission rate. We calculate and select 876 Bytes/637 ${\upmu }\mathrm{s}$ as the optimal payload length/payload transmission time. The full payload transmission time and frame lengths are listed in Table 1. We list the main simulation parameters in Table 2 and get results in Fig.2..

**Table 1:** Payload length and transmission time based on data rate (11 Mbps) for 40 nodes and 50 nodes.

40,50 nodes: payload length (Bytes)	Transmission time (${\upmu }\mathrm{s}$)
256	186
512	372
693	504
876	637
1023	744
2047	1488

**Table 2:** Main simulation parameters.

Parameters	Values	Parameters	Values
PHY header	192 bits	MAC header	224 bits
CTS length	304 bits	RTS length	352 bits
Slot time length	20 ${\upmu }\mathrm{s}$	DIFS length	50 ${\upmu }\mathrm{s}$
SIFS length	10 ${\upmu }\mathrm{s}$	EIFS length	364 ${\upmu }\mathrm{s}$
*m*	5	*K*	7
$W_0$	32 slots	$\lambda$	2

As shown in Fig. 2, the throughput (throughput-time) performance and network delay performance increase with the increase of payload transmission time, corresponding to the increase of payload length in the frame. With the increase of payload transmission time, the ratio curves of $S_V$ to network delay have the same change trend. They increase firstly to reach the peak value, and then decrease. The peak values appear at the same value of payload transmission time for network with different node numbers, which confirms the correctness of the analysis in this paper. Due to the lower collision probability, the data of 40 nodes are higher than that of 50 nodes in Fig. 2c,d, which means that throughput/(network delay) of 40 nodes is larger/(shorter) than that of 50 nodes.

The errors between the analysis results and the simulation results at 11 Mbps data rate are due to the cumulative errors caused by the separate analysis method of network delay or throughput-time. With larger values of payload transmission time than the peak value, the network delay has higher increase rate than the throughput-time does, although the throughput will increase with long payload length. The main reason for the higher increase rate of network delay may be the long judgment time of collision event. Based on the curves, we get two points: One is that there is a peak ratio of $S_V$ to network delay; The other is that the peak value is independent of node numbers and data rates, and its value is determined by the access mechanism parameters and propagation time.

### Results between RTS/CTS/DATA/ACK type and DATA/ACK type

**Figure 3:**
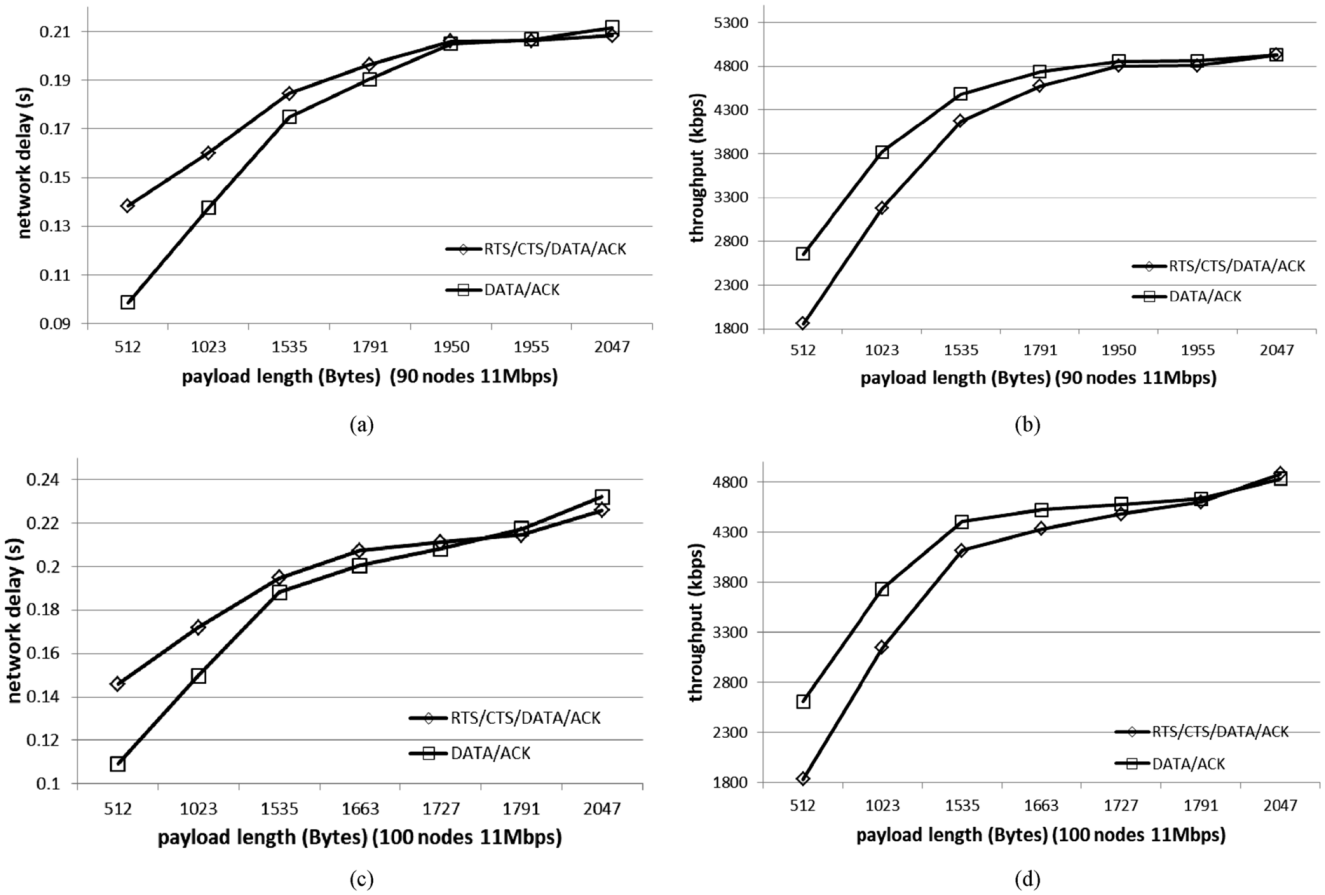
Network performance with DATA/ACK type and RTS/CTS/DATA/ACK type. In a network of 90 nodes, we get payload transmission time $h_t^{90}=1354 \, ({\upmu }s)$ and payload length $L_{payload}(h_t^{90})=1862 \, (\mathrm{Bytes})$ with data transmission rate 11 Mbps. In a network of 100 nodes, we get the payload length of 100 nodes $L_{payload}(h_t^{100})=1771 \, (\mathrm{Bytes})$, which is shorter than the payload length of 90 nodes $L_{payload}(h_t^{90})=1862 \, (\mathrm{Bytes})$. When it is smaller than the threshold $h_t$, the throughput performance and delay performance of DATA/ACK type are better than that of RTS/CTS/DATA/ACK type.

We can learn in (4) that the probability of busy channel *p* increases with the network nodes, and it means that more nodes lead to larger values of *p*. Due to $p=p_S + p_C$, the collision probability $p_C$ increases faster than the successful transmission probability $p_S$ with increase node number in the network. The more number of nodes there is, the lower the ratio of successful transmission probability to collision probability will be. With parameters in Table 2, we give the payload transmission time and payload length in Table 3. The simulated data are shown in Fig. 3.

**Table 3:** Payload Length of 90 nodes and 100 nodes with 11 Mbps.

90 nodes: payload length (Bytes)	Transmission time (${\upmu }\mathrm{s}$)	100 nodes: payload length (Bytes)	Transmission time (${\upmu }\mathrm{s}$)
512	372	512	372
1023	744	1023	744
1535	1116	1535	1116
1791	1302	1663	1209
1950	1418	1727	1256
1955	1422	1791	1302
2047	1488	2047	1488

As shown in Fig. 3, the network delay $E[D]^{RTS}$ of RTS/CTS/DATA/ACK type and the network delay $E[D]^{DATA}$ of DATA/ACK type increase with the increase of payload length in a frame. We show the simulated values [1950, 1955] (Bytes) ($H(D)=E[D]^{RTS}-E[D]^{DATA}$) in Fig. 3a for 90 nodes network. In this narrow range, the curve of $E[D]^{DATA}$ increases from smaller than that of $E[D]^{RTS}$ to larger than that of $E[D]^{RTS}$. Although the payload length (1950 (Bytes)) is longer than $L_{payload}(h_t)=1862 \,(Bytes)$ calculated in 90 nodes network, the error is minor. We also obtain similar results in Fig. 3c for 100 nodes network. There are two reasons: One is that the RTS/CTS frames can indeed reduce the collision duration of long payloads; The other is that the collision probability is high enough to exceed the overhead of the RTS/CTS frames exchange.

From above results, we can learn that the ratio $p_S/p_C$ of 100 nodes is lower than that of 90 nodes, which means that the threshold $L_{payload}(100 \ nodes)$ is smaller than the threshold $L_{payload}(90 \ nodes)$. The throughput performance of DATA/ACK type is better than that of RTS/CTS/DATA/ACK type when the payload transmission time is shorter than $h_t$. It can be inferred that when the data rates are higher (larger than 11 Mbps), the DATA/ACK type is better for data transmission, and the throughput performance and delay performance are better than that of RTS/CTS/DATA/ACK type. In WBN, the business data of blockchain technology exchanged at one time are not large, and the transmitted payload in one frame will not be too long. Because the throughput performance is not the only goal, we can analyze the network performance based on the payload transmission time in networks with different data rates. Based on the above analysis results, we can select the suitable payload length for better throughput performance and delay performance by DATA/ACK type.

## Conclusion

In WBN, delay performance is very important, and throughput performance is not the only goal. We have proposed an analytical model that the delay performance and throughput performance are equally important, which can be used to evaluate the network performance in networks with different data rates. We have separated the data transmission process from the business process of blockchain technology. We have proposed a ratio model of throughput-time to network delay. We have studied the performance between throughput and delay at different data transmission rates in WBN. We have obtained the optimal payload transmission time, which can be used for the optimization scheme of access parameters in WBN. Based on the DATA/ACK type, we have analyzed the different increase rates between the throughput-time and network delay with the increase of payload transmission time.

The data transmission process involves network status, throughput performance and delay performance. Communication throughput includes transaction throughput. Communication delay generally does not include transaction delay. Because the transaction delay contains an important processing delay, the processing delay is much longer than the communication delay. Based on the ratio model proposed in this paper, the performance of communication throughput and transaction throughput will be significantly affected if the communication delay includes or does not include transaction delay. The relevant conclusions in this work can be used to analyze consensus mechanisms and forks caused by delay, like in blockchain security and resource-limited network. For blockchain securities, especially the process level security, the delay reflects the network state and should further constrain (increase or decrease) the throughput performance. The constrained devices can send the computing requirements to the cloud, thereby constructing an access network based on the edge cloud. Next, we will focus our interest on the consensus mechanisms and blockchain securities.

## Data Availability

The datasets used and/or analysed during the current study available from the corresponding author on reasonable request.

## References

[1] Liu M, Yu FR (2019). Distributed resource allocation in blockchain-based video streaming systems with mobile edge computing. IEEE Trans. Wirel. Commun..

[2] Xu H, Klainea PV, Oniretia O (2020). Blockchain-enabled resource management and sharing for 6G communications. Digit. Commun. Netw..

[3] Li Y, Cao B, Liang L, Mao D, Zhang L (2021). Block access control in wireless blockchain network: design, modeling and analysis. IEEE Trans. Veh. Technol..

[4] Croman, K. *et al.* On scaling decentralized blockchains. In *2016 International Conference on Financial Cryptography Data Security*, 106–125 (2016).

[5] Ngubo CE, Dohler M (2020). Wi-Fi-dependent consensus mechanism for constrained devices using blockchain technology. IEEE Access.

[6] IEEE Standard for Information Technology-Telecommunications and Information Exchange Between Systems Local and Metropolitan area Networks Specific Requirements Part 11: Wireless LAN Medium Access Control (MAC) and Physical Layer (PHY) Specifications. IEEE Standards 802.11$$^{\rm T}$$M-2016 (2016).

[7] IEEE P802.11ax$$^{\rm T}$$M D6.0 (2019).

[8] Sun Y, Zhang L, Feng G (2019). Blockchain-enabled wireless Internet of Things: Performance analysis and optimal communication node deployment. IEEE Internet Things J..

[9] Abyaneh, A. Z., Zorba, N. & Hamdaoui, B. IEEE 802.11 ax based medium access design for wireless IoT-blockchain networks. In *GLOBECOM 2020-2020 IEEE Global Communications Conference*, 1–6 (IEEE, 2020).

[10] Cao B, Li M, Zhang L, Li Y, Peng M (2019). How does CSMA/CA affect the performance and security in wireless blockchain networks. IEEE Trans. Ind. Inform..

[11] Chen, S., Zhang, J., Shi, R., Yan, J. & Ke, Q. A comparative testing on performance of blockchain and relational database: Foundation for applying smart technology into current business systems. In *2018 International Conference on Distributed, Ambient, Pervasive Interactions*, 21–34 (2018).

[12] Bianchi G (2000). Performance analysis of the IEEE 802.11 distributed coordination function. IEEE J. Sel. Areas Commun..

[13] Dai L, Sun X (2013). A unified analysis of IEEE 802.11 DCF networks: Stability, throughput, and delay. IEEE Trans. Mob. Comput..

[14] Chun S, Xianhua D, Pingyuan L, Han Z (2012). Adaptive access mechanism with optimal contention window based on node number estimation using multiple thresholds. IEEE Trans. Wirel. Commun..

[15] Kosek-Szott K, Rapacz N (2019). Tuning wi-fi traffic differentiation by combining frame aggregation with txop limits. IEEE Commun. Lett..

[16] Lin, Y. & Wong, V. W. WSN01-1: Frame aggregation and optimal frame size adaptation for IEEE 802.11 n WLANs. In *2006 IEEE Globecom*, 1–6 (2006).

[17] Sharon O, Alpert Y (2017). Single User MAC Level Throughput Comparision: IEEE 802.11 ax vs. IEEE 802.11 ac. Wirel. Sens. Netw..

[18] Vijay BT, Malarkodi B (2019). MAC improvements for very high throughput WLANs. Int. J. Commun. Netw. Distrib. Syst..

[19] Kosek-Szott K, Cuka G (2017). Consequences of performing DL MU-MIMO transmissions with TXOP sharing for QoS provisioning in IEEE 802.11 ac networks. IEEE Commun. Lett..

[20] Seytnazarov S, Choi JG, Kim YT (2018). Enhanced mathematical modeling of aggregation-enabled WLANs with compressed blockACK. IEEE Trans. Mob. Comput..

[21] Mansour K, Jabri I, Ezzedine T (2019). Revisiting the IEEE 802.11 n A-MPDU retransmission scheme. IEEE Commun. Lett..

[22] Hwang HY (2020). Analysis of throughput and delay for an underwater Multi-DATA train protocol with multi-RTS reception and block ACK. Sensors.

[23] Sakurai T, Vu HL (2007). MAC access delay of IEEE 802.11 DCF. IEEE Trans. Wirel. Commun..

[24] Danzi P, Kalr AE, Stefanovic C, Popovski P (2019). Delay and communication tradeoffs for blockchain systems with lightweight IoT clients. IEEE Internet Things J..

[25] Li, C. *et al.* A decentralized blockchain with high throughput and fast confirmation. In *2020 USENIX Annual Technical Conference (USENIXATC 20),* 515–528 (2020).

[26] Eyal, I., Gencer, A. E., Sirer, E. G. & Van Renesse, R. Bitcoin-NG: A scalable blockchain protocol. In *2016 13th USENIX Symposium on Networked Systems Design Implementation (NSDI 16)*, 45–59 (2016).

[27] Wang, J. & Wang, H. Monoxide: Scale out blockchains with asynchronous consensus zones. In *2019 16th USENIX Symposium on Networked Systems Design and Implementation (NSDI 19)*, 95–112 (2019).

[28] Leonardos S, Reijsbergen D, Piliouras G (2020). Weighted voting on the blockchain: Improving consensus in proof of stake protocols. Int. J. Netw. Manag..

[29] Huang, Y., Zeng, Y., Ye, F. & Yang, Y. Incentive assignment in PoW and PoS hybrid blockchain in pervasive edge environments. In *2020 IEEE/ACM 28th International Symposium on Quality of Service (IWQoS)*, 1–10 (IEEE, 2020).

[30] Bruno, R., Conti, M., & Gregori, E. IEEE 802.11 Optimal Performances: RTS/CTS Mechanism vs. Basic Access. In *2002 13th IEEE International Symposium on Personal, Indoor and Mobile Radio Communications*, 1747–1751 (IEEE, 2002).

[31] Edalat, Y., Obraczka, K. & Amiri, B. A machine learning approach for dynamic control of RTS/CTS in WLANs. In *2018 proceedings 15th EAI International Conference on Mobile Ubiquitous Systems: Computing, Networking Services*, 432–442 (2018).

[32] Chatzimisios, P., Boucouvalas, A. C. & Vitsas, V. Optimisation of RTS/CTS handshake in IEEE 802.11 Wireless LANs for maximum performance. In *IEEE Global Telecommunications Conference Workshops 2004*, 270–275 (IEEE, 2004).

[33] Yin, Y., Gao, Y., Manzoor, S. & Hei, X. Optimal RTS threshold for IEEE 802.11 WLANs: basic or RTS/CTS? In *2019 IEEE SmartWorld, Ubiquitous Intelligence & Computing, Advanced & Trusted Computing, Scalable Computing & Communications, Cloud & Big Data Computing, Internet of People and Smart City Innovation (SmartWorld/SCALCOM/UIC/ATC/CBDCom/IOP/SCI)*, 1620–1625 (IEEE, 2019).

[34] Islam, M. M. & Zhang, Z. An implementation of RTS/CTS mechanism and CS algorithm for maximum performance of D2D communication. In *2019 IEEE 11th International Conference on Communication Software and Networks (ICCSN)* 523–528 (IEEE, 2019).

[35] Baiocchi, A., Garlisi, D., Santaromita, G. & Tinnirello, I. Moving RTS/CTS to the frequency domain: An efficient contention scheme for 802.11 ax networks. In *2019 31st International Teletraffic Congress (ITC 31)* 140–148 (2019).

[36] Guo, Y., Li, Z., Lv, R. & Yang, Z. Evaluating the impact of aggregation and RTS/CTS schemes on IEEE 802.11 based linear wireless ad-hoc networks. *Authorea Prepr.* 1–17 (2021).

[37] Singh, B. & Mishra, R. Performance analysis of IEEE 802.11 b DCF-basic access and RTS/CTS in CBTC under field conditions. In *2019 6th International Conference on Signal Processing and Integrated Networks (SPIN)*, 562–567 (2019).

[38] Leng, J. *et al.* Blockchain security: A survey of techniques and research directions. *IEEE Trans. Serv. Comput.* **15**(4), 2049–2510, (2022).

[39] Wang, W. *et al.* Blockchain-based reliable and efficient certificateless signature for IIoT devices. *IEEE Trans. Ind. Inform.* **18**(10), 1551–3203 (2022).

[40] Deebak, B. D. *et al.* Lightweight blockchain based remote mutual authentication for AI-empowered IoT sustainable computing systems. *IEEE Internet Things J.*10.1109/JIOT.2022.3152546 (2022).

[41] Deebak, B. D., Memon, F. H., Dev, K., Khowaja, S. A., Wang, W. & Qureshi, N. M. F. TAB-SAPP: A trust-aware blockchain-based seamless authentication for massive IoT-enabled industrial applications. *IEEE Trans. Ind. Inform.*10.1109/TII.2022.3159164 (2022).

